# Echoic Sensory Substitution Information in a Single Obstacle Circumvention Task

**DOI:** 10.1371/journal.pone.0160872

**Published:** 2016-08-05

**Authors:** Andrew J. Kolarik, Amy C. Scarfe, Brian C. J. Moore, Shahina Pardhan

**Affiliations:** 1 Centre for the Study of the Senses, Institute of Philosophy, University of London, London, United Kingdom; 2 Vision and Eye Research Unit (VERU), Postgraduate Medical Institute, Anglia Ruskin University, Cambridge, United Kingdom; 3 Department of Psychology, University of Cambridge, Cambridge, United Kingdom; 4 Department of Clinical Engineering, Medical Imaging and Medical Physics Directorate, Sheffield Teaching Hospitals NHS Foundation Trust, Sheffield, United Kingdom; University of Bath, UNITED KINGDOM

## Abstract

Accurate motor control is required when walking around obstacles in order to avoid collisions. When vision is unavailable, sensory substitution can be used to improve locomotion through the environment. Tactile sensory substitution devices (SSDs) are electronic travel aids, some of which indicate the distance of an obstacle using the rate of vibration of a transducer on the skin. We investigated how accurately such an SSD guided navigation in an obstacle circumvention task. Using an SSD, 12 blindfolded participants navigated around a single flat 0.6 x 2 m obstacle. A 3-dimensional Vicon motion capture system was used to quantify various kinematic indices of human movement. Navigation performance under full vision was used as a baseline for comparison. The obstacle position was varied from trial to trial relative to the participant, being placed at two distances 25 cm to the left, right or directly ahead. Under SSD guidance, participants navigated without collision in 93% of trials. No collisions occurred under visual guidance. Buffer space (clearance between the obstacle and shoulder) was larger by a factor of 2.1 with SSD guidance than with visual guidance, movement times were longer by a factor of 9.4, and numbers of velocity corrections were larger by a factor of 5 (all *p*<0.05). Participants passed the obstacle on the side affording the most space in the majority of trials for both SSD and visual guidance conditions. The results are consistent with the idea that SSD information can be used to generate a protective envelope during locomotion in order to avoid collisions when navigating around obstacles, and to pass on the side of the obstacle affording the most space in the majority of trials.

## Introduction

A requirement of travelling through urban environments is the ability to safely navigate around obstacles [1]. Visual loss substantially reduces the spatial information available for safe locomotion, although aids such as canes and guide dogs can partially compensate for this. Echoes from self-generated sound (for reviews, see [2–4]), or from electronic travel aids called sensory substitution devices (SSDs), which convey spatial information via an unimpaired modality [5], can also help visually impaired individuals to circumvent obstacles. The current study addressed three questions: 1) Can SSD information be used to create a ‘protective-envelope’, or buffer space, to help protect people during visionless navigation? 2) What are the exact kinematics of obstacle circumvention using an echoic SSD, and how do they compare to those for echolocation-guided locomotion, as measured previously [6]? 3) Do blindfolded participants using an SSD pass on the side of an obstacle affording the most space, as they typically do when visual information is available?

Previous work has investigated obstacle avoidance under visual guidance [7], and using sensory substitution information when vision is absent [8–11]. Some SSDs convert visual information into an auditory or a haptic signal using a predetermined transformation algorithm [12]. Such SSDs, referred to here as visual pattern SSDs, include the vOICe (the central letters stand for “oh I see,” [13–15]), and the “Prosthesis Substituting Vision with Audition” (PSVA, [16, 17]). Other SSDs, referred to here as echoic SSDs, work on an echolocation principle, and include Kay’s Advanced Spatial Perception Aid (KASPA, [18]) and the Miniguide [19, 20]. Using an ultrasound source and a receiver, an echoic SSD detects signal echoes, which are used to calculate the distance to an obstacle using the time delay between the emission and echo. This information is then converted into a haptic or auditory signal.

A number of studies have demonstrated that echoes from self-generated sounds can be used to perceive the spatial location of an obstacle, and to approach it as closely as possible without touching it, for both blind [21–24] and blindfolded sighted participants [22, 25, 26]. Spatiotemporal flow fields from echoic SSDs can also provide information regarding the layout of the environment to inform safe locomotion. Maidenbaum et al. [8] showed that blindfolded-sighted participants were able to use an echoic SSD (Eyecane) for distance estimation, navigation and obstacle detection. Chebat et al. [10] showed that blind participants and blindfolded-sighted controls could use the Eyecane to navigate through real and virtual mazes. In virtual environments, the increased sensory range of 5 m provided by a virtual Eyecane allowed participants to take shorter navigation paths and make fewer collisions than when a virtual white cane was used [9].

Although SSDs can be highly useful as a navigation aid, collisions do sometimes occur. Levy-Tzedek et al. [27] reported that the rate of collisions was higher using the Eyecane SSD than using vision to navigate in virtual mazes. Veraart et al. [28] reported that binocularly blinded cats were able to use echoic SSD information to assess depth in a jumping task and to avoid obstacles when moving through a maze. Veraart and Wanet-Defalque [29] showed that use of an echoic SSD by early-blind humans increased performance for judging the distance and direction of obstacles located along various routes. Hughes [30] reported that echoic SSD information allowed blindfolded normally sighted participants to judge whether narrow apertures were passable in the majority of trials. Kolarik et al. [20] showed that echoic SSD information allowed blindfolded participants to make the shoulder rotations needed to move through narrow apertures. As expected, compared to visual guidance, shoulder rotations made under SSD guidance were greater, movement times were longer, and collisions sometimes occurred. Hicks et al. [11] developed a depth-based visual display as an assistive device for people with residual vision. Sighted participants were able to use the device to navigate an obstacle course, and partially sighted individuals were able to respond to illuminated objects presented to their residual visual fields using the device.

Chebat et al. [31] showed that blind and normally sighted blindfolded participants were able to navigate an obstacle course using a visual pattern SSD called the Tongue Display Unit (TDU). Blind and sighted participants successfully avoided large obstacles on approximately 78% and 71% of trials, respectively. Kolarik et al. [6] showed that echoes from sounds that were self-generated by sighted blindfolded participants could effectively guide locomotion around an obstacle in the majority of trials. Performance was evaluated by successful avoidance as well as using 3-dimensional motion capture to quantify various indices including buffer space (the clearance between the shoulders and the obstacle, following Franchak et al. [32], who used the term buffer space to indicate the margin between the body and sides of an aperture during locomotion). Compared to visual guidance, buffer space, movement times, and the number of velocity corrections were greater when using echolocation.

Under visual navigation, people appear to generate a protective envelope during locomotion that allows sufficient time and distance to perceive hazards in the local environment, and plan gait adaptations to avoid collisions [33]. If SSD information can be used to generate a protective envelope during visionless navigation, then under SSD guidance participants should be able to navigate around an obstacle on the majority of trials without collision and they should do this in an efficient manner once the obstacle has been detected. We assessed whether this was the case. Locomotion under visual guidance was measured to provide a baseline for comparison. The experiment was designed to allow comparison with a previous study [6] using echoes from self-generated mouth clicks, as well as a no-click/no-vision condition in which participants wore blindfolds and did not produce mouth clicks, as the task, procedure, and kinematics measured were similar to those used by Kolarik et al. [6].

In their review of the effectiveness of electronic mobility devices, Roentgen et al. [34] noted that although existing studies showed that the use of SSDs by those with visual impairment was generally beneficial, no standardized methods or existing measurement instruments were used in the studies that were reviewed (apart from one study that reported preferred walking speed). Kinematic measures of SSD-guided obstacle circumvention have not been obtained in previous studies, and an examination of the kinematics would be relevant to blindness and rehabilitation training, providing objective assessments of how practical and efficient SSD information is for everyday navigation. Although previous work has shown that SSDs can help prevent collision with obstacles [31], other important practical information is lacking, including an assessment of the time needed to scan and safely navigate around an obstacle using an SSD and the size of the buffer space. Reduced walking speed, indicated by increased movement time, is also an important safety mechanism, since if a collision does occur it is less likely to result in injury. Previous research [35] demonstrated that participants with substantially reduced visual fields slowed down and increased the clearance between the obstacle and body when navigating around multiple obstacles. The participants in that study appeared to optimize safety (collision avoidance) at the cost of spending more energy (greater clearance).

The measure “velocity corrections” was chosen for the present study to characterize the fluidity of movement and navigation around an obstacle. If participants navigating under SSD guidance frequently stop and start, this would have implications for the practicality of use in non-laboratory settings, and may also affect energy consumption. In addition, we investigated whether participants passed obstacles on the side affording the most space under SSD guidance, as has been previously shown for vision [7]. We also measured initial path deviations (the distance from the obstacle where participants first deviated from moving in a straight line), indicative of path planning. Kinematic information may highlight possible limitations in the practicality of SSDs outside a research setting [36]. In summary, the current study measured for the first time the kinematics of SSD-guided single-obstacle circumvention. The kinematics were directly compared to those for echolocation-guided obstacle circumvention, as measured in a previous study [6].

We hypothesized that echoic SSD information would enable blindfolded normally sighted participants to safely navigate around a single obstacle in the majority of trials, passing on the side affording the most space. Navigation under SSD guidance was hypothesized to be less accurate than in a visual baseline condition, indicated by a greater number of collisions and velocity corrections, larger buffer space, and longer movement times.

## Methods

### Participants

12 participants took part (8 males and 4 females, mean age 31 yrs, range 21–42 yrs). All had normal or near-normal hearing, defined as better-ear average (BEA) hearing thresholds across the frequencies 500, 1000, 2000 and 4000 Hz ≤25 dB HL, as measured using an Interacoustics AS608 audiometer. All participants reported normal or corrected-to-normal vision. None of the participants reported having any prior experience with SSDs. The experiments followed the tenets of the Declaration of Helsinki. Written informed consent was obtained from all participants following an explanation of the nature and possible consequences of the study. The experiments were approved by the Anglia Ruskin University Ethics committee.

### Apparatus and data acquisition

**Fig 1:**
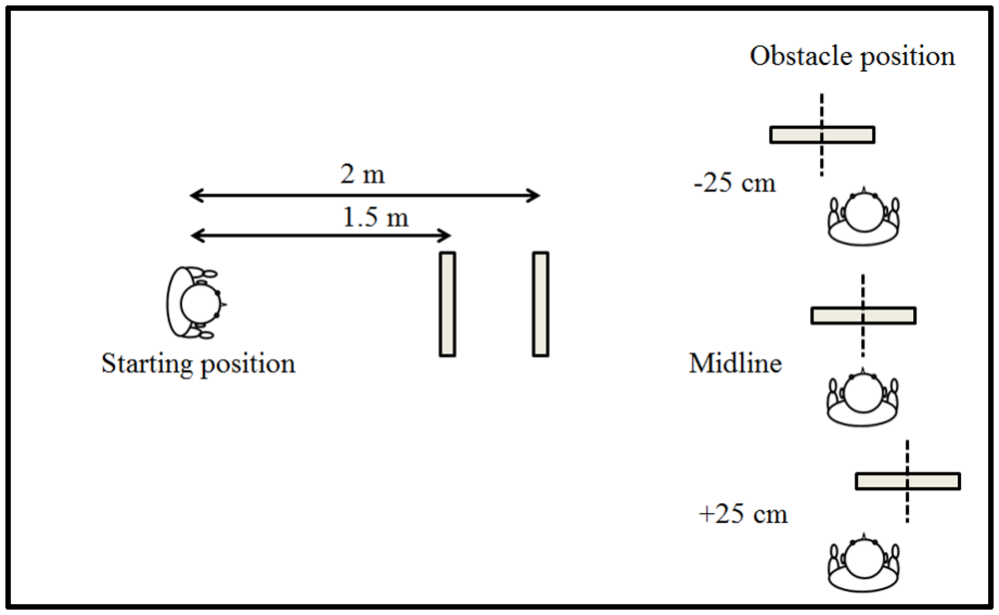
Two views of the layout for obstacle circumvention. The 0.6-m wide obstacle was either straight ahead on the midline relative to the participant, or ±25 cm to the left or right (right part of figure), at an approach distance of either 1.5 or 2 m (left part of figure).

Testing took place in a quiet room measuring 5.7 × 3.5 m with a ceiling height of 2.8 m, with an ambient sound level of approximately 36 dBA. The floor was carpeted, the walls were painted, and the ceiling was tiled. The obstacle measured 0.6 (width) × 2 m (height). It was constructed of wood and was covered by smooth aluminium foil, following Arnott et al. [37], to achieve high reflectivity. It was movable, flat and rectangular with a thickness of 0.6 cm, with a small plastic triangular frame at the bottom on the side away from the participant, mounted on castors. See Fig 1 for a schematic of the experimental layout. The obstacle was positioned in the approximate center of the room to minimize SSD reflections from surfaces other than the obstacle. The experimenter and participants maintained silence during testing.

The SSD used in the study was the Miniguide, manufactured by GDP Research [19, 20]. The device emitted an ultrasound signal. The distance between the device and the obstacle was estimated from the time taken for the emitted signal to reflect back from the obstacle to a receptor on the device. Spatial information was provided via a tactile vibration signal; the rate of vibration was proportional to the distance between the device and the obstacle. The range of the device (the distance over which an obstacle could be detected) was set to 1 m [20]. The device was held in the participant’s dominant hand. Participants were instructed to tuck their elbow against their side when using the SSD to prevent arm movements in an anterior/posterior direction and to ensure that the hand holding the device was perpendicular to the body, in order to standardize the SSD feedback received across the trials without any influence from the movements of the arm [20].

The experimenter recorded the trials when a collision occurred between any part of the participant’s body or the SSD and the obstacle, and whether the participant passed the obstacle on the left or right side. An 8-camera motion capture system (Vicon Bonita; Oxford Metrics Ltd) collected 3-D kinematic data at 50 Hz. Retro-reflective spherical markers were attached bilaterally to the participant at the following anatomical locations: the antero-lateral and postero-lateral aspects of the head, the most distal, superior aspect of the 1st toe, the most distal, superior aspect of the 5th toe, the posterior aspect of the calcanei, and the acromio-clavicular joint. A marker was also placed on the posterior aspect of the dominant hand and another on the sternum. In order to define the obstacle within the Vicon coordinate system, three markers were attached to the front aspect of the obstacle. Marker trajectory data were filtered using the cross validatory quintic spline smoothing routine, with ‘smoothing’ options set at a predicted mean-squared error value of 10 and processed using the Plug-in gait software (Oxford Metrics). Although reflections from the aluminium foil covering the obstacle were sometimes recorded by the Vicon system, care was taken to label each marker individually for each trial to exclude these erroneous reflections from the analyses.

Kinematic variables for the obstacle circumvention task were assessed using custom written Visual Basic scripts (Table 1). A velocity correction was deemed to occur when the participant accelerated or decelerated in the anterior/posterior direction. To avoid including very small fluctuations in velocity, this was required to occur for the duration of 50 frames (1 sec). Stopping and starting were counted as velocity corrections, as were slowing down and speeding up. A change in trajectory was not deemed to be a velocity correction unless it involved a forward acceleration/deceleration. If the participant took a side step without slowing down in the anterior/posterior direction then this was not counted. However if they stopped, took a side step and then continued forward again, this would count as two velocity corrections (1 for decelerating prior to the side step, and 1 for accelerating after the side step).

**Table 1:** Kinematic variables assessed for the single obstacle circumvention task.

Variable	
Buffer space	The medio-lateral distance between the shoulder marker and obstacle, at the point of passing the obstacle, specified as the point where the shoulder marker passed the marker attached to the front aspect of the obstacle.
Movement time	The time taken to complete the movement, measured from when the sternum marker was 1 m from the obstacle in the anterior-posterior direction, until the point of passing the obstacle marker (or the spatial position of the virtual obstacle marker for ‘no obstacle’ catch trials).
Velocity corrections	The number of changes in velocity i.e. changes from acceleration to deceleration or vice versa, measured from when the sternum was 1 m away from the obstacle until the point of passing the obstacle marker (or the spatial position of the virtual obstacle marker for ‘no obstacle’ catch trials). See main text for additional information.
Initial path deviation	The distance between the obstacle marker and the sternum marker when the final lateral change of direction occurred prior to the trajectory of the sternum marker departing from that for the average of the obstacle-absent trials by more than ±1 standard deviation (SD, measured within participants).

### Procedures

There were two locomotion guidance conditions: SSD and full vision. Following previous studies [6, 20], the vision condition was performed second to avoid ‘training’ the participant with regard to the range of distances and obstacle positions tested.

The SSD condition consisted of a training phase followed by a testing phase. Previous studies that investigated the use of echoic SSDs for passing through apertures trained participants for approximately 5 minutes [30, 38]. We provided more extensive training than used in these studies; participants practiced navigating around the obstacle using the SSD from an approach distance of 1.75 m for a minimum of 15 minutes. For the first 5 minutes the participants were allowed to keep their eyes open, for the next five minutes they were encouraged to close their eyes, and for the last five minutes they were blindfolded [6, 20]. In the first 10 minutes, the obstacle was placed straight ahead in front of the participant, and for the last 5 minutes the location of the obstacle was varied randomly among three positions: straight ahead and 25 cm to the participants’ left or right.

In the testing phase, participants were blindfolded and instructed to maintain a straight line of travel until the obstacle was detected using the device and then to circumnavigate around the obstacle without collision. Participants were told that the obstacle would sometimes be absent, and in this case they should maintain a straight line of travel until the experimenter instructed them to stop. Trials were terminated when the participant had successfully moved past the obstacle, when a collision occurred, or, for trials where the obstacle was absent, when they moved more than 2 m forward from the starting position. Participants commenced each trial from the same starting position, and the obstacle was initially 1.5 m from the participant for half of the trials and 2 m away for the other half, selected randomly. These distances were shorter than typically used for visual obstacle circumvention tasks (e.g. 5 m [1, 7]), as pilot data showed that participants often stopped a short distance in front of the obstacle and then explored it using the SSD, so a longer distance was not required. This has also been shown for locomotion through apertures using an SSD [20], or using echolocation to avoid an obstacle [6]. The obstacle was randomly varied in lateral location relative to the participant (midline, or 25 cm to the right or left), with 3 repetitions for each obstacle location, and 6 ‘no obstacle’ catch trials. In total, each participant completed 24 trials.

The full vision condition consisted of a testing phase only, similar to previous studies of visually guided navigation around an obstacle [6, 7]. This was the same as for the SSD testing phase, except that participants were blindfolded between trials only and they removed the blindfold at the start of each trial when signaled by the experimenter.

For both conditions, before each trial, the participants’ feet were aligned against a removable plastic box so that they faced directly forward, to ensure that they would walk straight ahead (this was checked by the experimenter). A shoulder tap from the experimenter signaled the beginning of the trial. Once completed, the experimenter led participants back to the starting point, and stood in the same place to the side for the duration of each trial. Except when navigating under visual guidance, participants wore close-fitting blindfolds, and their ears were occluded using headphones with sound-attenuating muffs. Participants were not allowed to use their hands to touch the obstacle and no feedback was provided during the testing phase. The sound-attenuating headphones prevented participants from hearing the obstacle being moved (this was checked during pilot testing; participants were asked to report whether the obstacle was present or had been moved at the start of each trial while blindfolded. None reported being able to do this). The experiment took approximately 1.5 hours to complete for each participant.

### Statistical analyses

Unless otherwise stated, repeated-measures analyses of variance (ANOVAs) were used to analyze how the buffer space, the side of obstacle chosen (right or left), the overall movement time, and the number of velocity corrections were affected by guidance condition (SSD and vision), obstacle lateral location (left, midline, or right relative to the participant), and repetition (trial 1–3). The significance level was chosen as *p*<0.05. As preliminary analyses indicated that scores for all measures were not significantly different for the two approach distances (*p*>0.05), the results for these were pooled. Proportional data for side of avoidance were subjected to arcsine transformation prior to analysis, as recommended by Howell [39]. Post hoc analyses were performed using Bonferroni correction.

## Results

The proportions of obstacle-present trials for which no collisions occurred under SSD guidance were 0.90, 0.96, and 0.92 for obstacles located to the left, midline and right, respectively. No collisions occurred under visual guidance. See S1 File for participant data. The proportion of successful obstacle circumventions under SSD and visual guidance was substantially greater than for the no-click/no-vision condition of Kolarik et al. [6], for which the proportion of obstacle-present trials when no collisions occurred was 0.22, 0.22 and 0.06, for obstacles located to the left, midline and right, respectively. The results of Kolarik et al. [6] suggest that under conditions of no-click/no-vision guidance participants had severe difficulty even detecting the presence of the obstacle, and were not able to pass the obstacle on the side affording the most space or to generate a protective envelope to avoid collision in the majority of trials.

**Fig 2:**
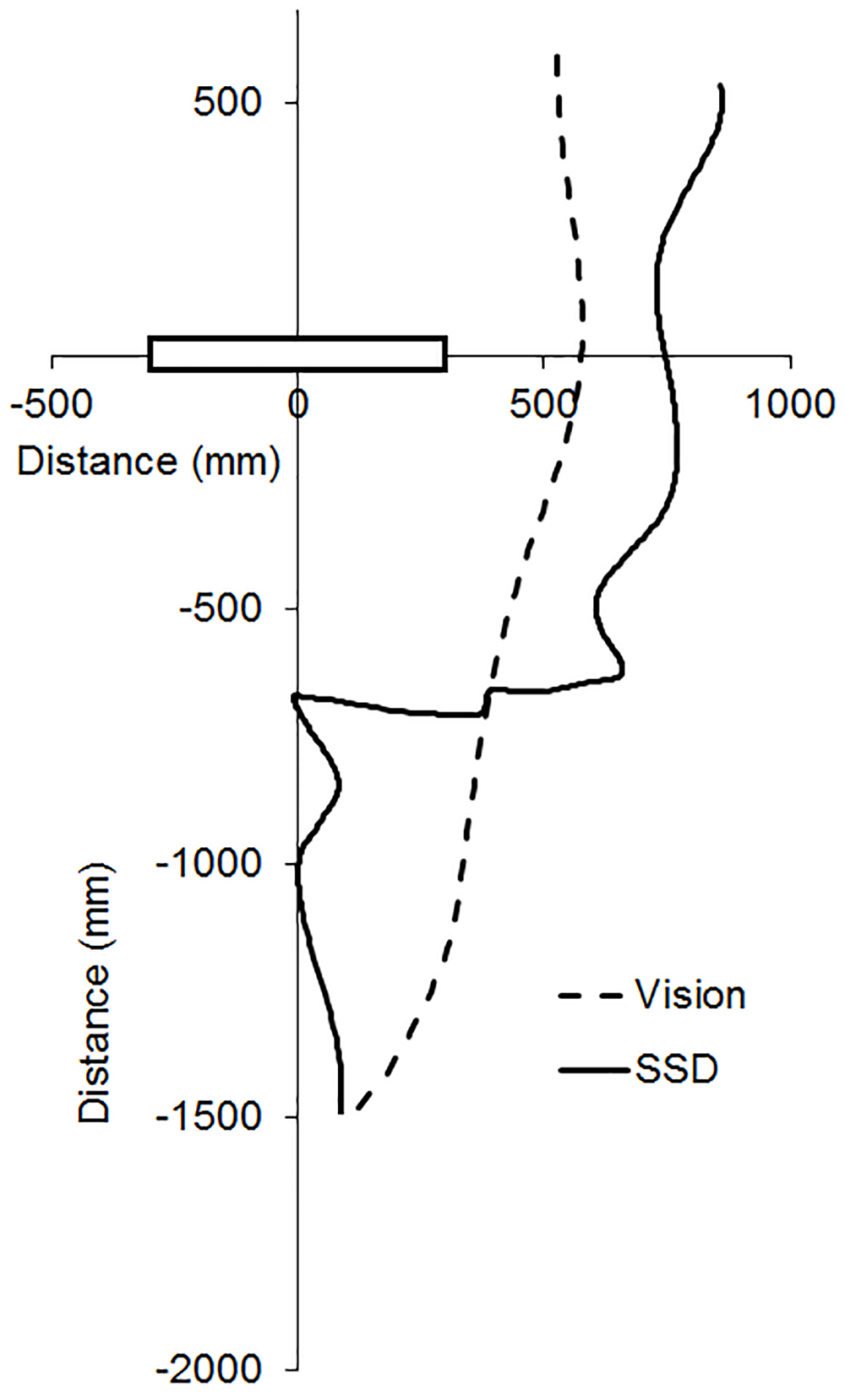
Representative participant trajectories under visual guidance (dashed line) and SSD guidance (solid line) when circumventing an obstacle (black rectangle). Data are shown for the left shoulder marker, for a participant approaching the obstacle from a distance of 1.5 m. The obstacle’s lateral location was 25 cm to the left of the participant.

Fig 2 shows trajectories for a representative participant under visual guidance (dashed line) and SSD guidance (solid line). The obstacle was 25 cm to the left (black rectangle). Under SSD guidance, participants showed distinct deviations from the straight-ahead direction only when the obstacle was within the operating range of the SSD (1 m from the obstacle), whereas under visual guidance, participants deviated from straight ahead immediately upon initiating their movement. Under SSD guidance, the mean distances from the obstacle at which participants deviated by more than ± 1 SD from obstacle absent direction (with standard errors in parentheses) were 752 (46), 776 (51), and 782 (59) mm for the left, midline, and right obstacles, respectively. Under both visual and SSD guidance participants moved roughly straight ahead in no-obstacle catch trials, indicating that they only deviated markedly from straight ahead when an obstacle was present.

**Fig 3:**
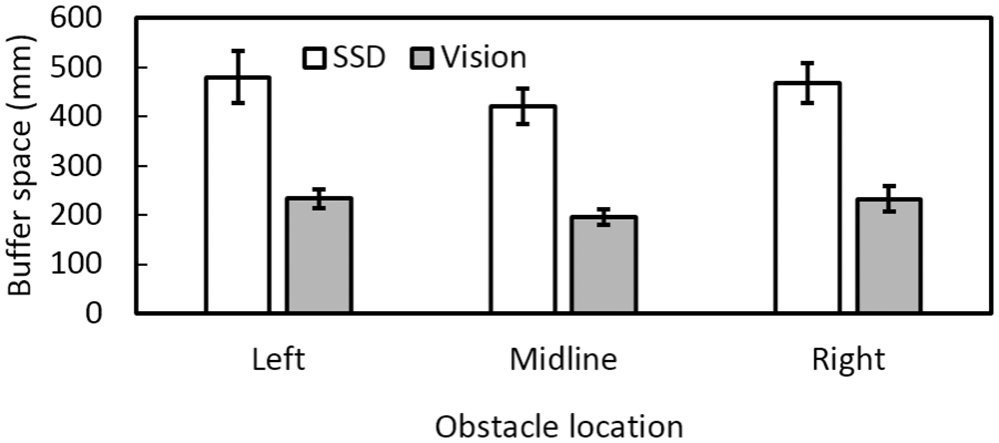
Mean buffer space between the shoulder and obstacle at the point of passing the obstacle, for each obstacle location. Open bars show buffer space under SSD guidance and grey bars show buffer space using vision. Here and in subsequent figures error bars denote ±1 standard error.

**Fig 4:**
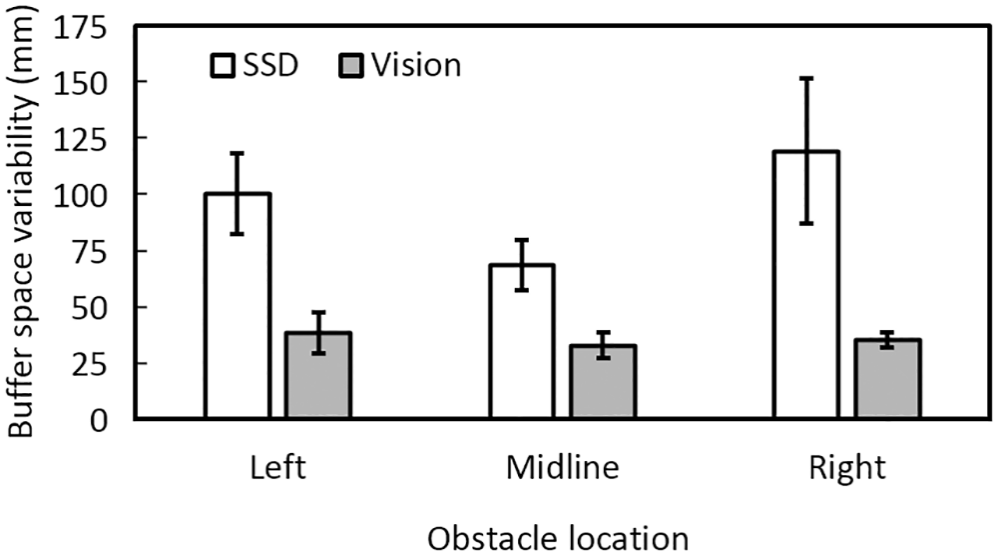
Mean buffer space variability under SSD guidance (open bars) and visual guidance (grey bars) for each obstacle location.

Fig 3 shows that for obstacle-present trials on which collisions did not occur, buffer space was greater under SSD guidance than under visual guidance for all obstacle positions. The mean buffer spaces under SSD guidance were 480, 422, and 468 mm for the left, midline, and right obstacles, respectively, while those under visual guidance were 233, 196, and 233 mm, respectively. There was a main effect of guidance condition [*F*(1, 11) = 42.4, *p* = 0.001] but no effect of obstacle location. To assess the variability of the buffer space, the SD of the buffer space across trials was calculated for each participant and each condition, and then the SDs were averaged across participants for each condition. Fig 4 shows the variability estimated in this way under SSD and visual guidance. Variability under SSD guidance (100, 69, and 119 mm for the left, midline, and right obstacles, respectively) was greater than under vision (39, 33, and 35 mm, respectively), suggesting that participants were less accurate in judging the position of the nearest edge of the obstacle under SSD guidance than under vision.

**Fig 5:**
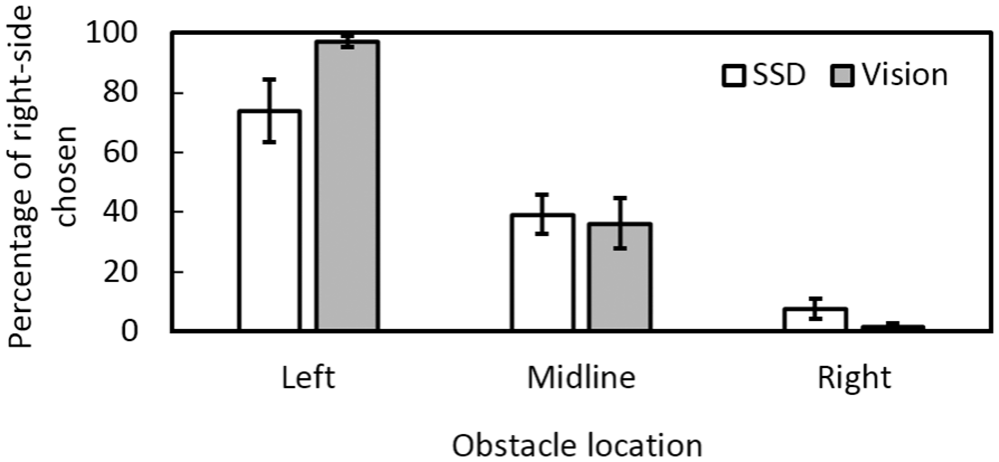
Percentage of times the right side was chosen for each obstacle location under SSD guidance (open bars) and visual guidance (grey bars) for each obstacle location.

Under both SSD and visual guidance, participants generally passed the obstacle on the side that afforded the most space. In other words, participants mostly passed on the right side when the obstacle was positioned to the left, and passed on the left side when the obstacle was positioned to the right (Fig 5). The data for the side that was chosen showed a main effect of obstacle position (*F*(2, 22) = 75.01, *p* = 0.001), but not guidance condition (*F*(1, 11) = 0.58, *p* = 0.46), and a significant interaction between obstacle position and guidance condition (*F*(2, 22) = 7.95, *p* = 0.003). In both conditions, the right side was chosen significantly more often when the obstacle was on the left than when it was on the midline or the right, and when the obstacle was on the midline than when it was on the right (all *p* < 0.01).

**Fig 6:**
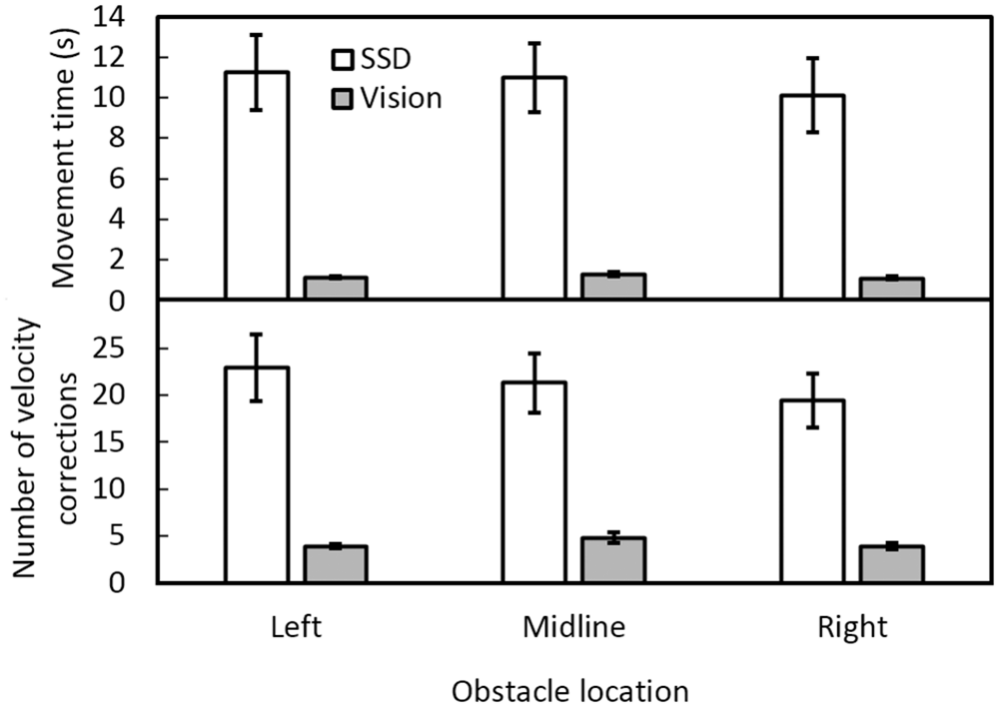
Mean movement times (upper panel) and numbers of velocity corrections (lower panel) under SSD guidance (open bars), and visual guidance (grey bars) for each obstacle location.

The mean movement times to circumvent the obstacle, and the mean number of velocity corrections (Fig 6, upper and lower panels respectively) were larger with the SSD than with vision. When navigating using the SSD, the mean movement times were 11, 11 and 10 s for obstacles located to the left, midline and right, respectively, compared to 1.1, 1.2, and 1.1 s, respectively, for vision. There was a main effect of guidance condition [*F*(1, 11) = 28.96, *p* = 0.001], but no effect of obstacle location. When navigating using the SSD, the mean numbers of velocity corrections were 23, 21, and 20 for obstacles that were located to the left, midline and right, respectively, while using vision they were 4, 5, and 4, respectively. There was a significant main effect of guidance condition [*F*(1, 11) = 31.05, *p* = 0.001], but not of obstacle location.

To investigate whether there was any reduction in movement speed simply due to being blindfolded, regardless of obstacle circumvention, movement times were assessed for no-obstacle catch trials. The mean movement times were 1, 5, and 8 s when navigating using vision, SSD guidance, and in the no-click/no-vision condition of Kolarik et al. [6], respectively. The mean numbers of velocity corrections were 4, 9, and 20 using vision, the SSD and in the no-click/no-vision condition, respectively. These findings suggest that participants moved more slowly when using the SSD than when using vision mainly because of the absence of vision, rather than because of SSD use per se.

## Discussion

The results show that: 1) echoic SSD information guided locomotion by blindfolded participants in a single-obstacle circumvention task with 93% accuracy; 2) Buffer space using the SSD was larger by a factor of 2.1 than for visual guidance; 3) Movement times were longer by a factor of 9.4, and number of velocity corrections were larger by a factor of 5 for SSD than for visual guidance; 4) Using SSD information, participants generally passed the obstacle on the side affording the most space. These results can be compared to those for a previous study in which blindfolded, normally sighted, non-expert echolocators performed a similar task using echolocation to guide their movements around an obstacle [6]. In that study, participants circumvented the obstacle without collision in 67% of the trials. Compared to visual guidance, the buffer space was larger by a factor of 1.8, movement times were longer by a factor of 27, and number of velocity corrections was larger by a factor of 14. Also, in that study participants did not generally pass on the side of the obstacle affording the most space. The results suggest that for blindfolded participants, locomotion using an echoic SSD is more accurate and faster than when using echolocation based on self-generated sounds. Further investigation is needed to test the magnitude of deviation from the midline required for participants to choose the path affording most space when using SSD guidance.

The current findings are consistent with the idea that echoic SSD information enables a protective envelope to be generated during locomotion, similar to that generated using visual information. The buffer space was larger under SSD guidance than under visual guidance. One possible reason for this is that the wavelength of ultrasound imposes a limit on the spatial detail available from echoic SSDs [40, 41]. The distance and location information available with the SSD were less precise than provided by vision, and participants may have allowed for this (perhaps without conscious awareness) by increasing the size of the protective envelope under SSD guidance. Consistent with this, the measure of variability of the buffer space was greater under SSD guidance than under vision. Another possibility is that the size of the protective envelope was not altered by the perceptual system, but buffer space increased due to misjudging the obstacle location or overestimating the obstacle width. A third possibility is that the increased buffer space under SSD guidance was partly due to increased postural sway, since the participants were blindfolded. Franchak et al. [32] described how a larger buffer space may be produced either when the participant is exercising greater caution to avoid high speed collision or when lateral sway increases, requiring larger spatial buffers for passage.

One potential limitation of our study arises from the possible obstacle locations being fixed. Participants may have learned these locations during the last part of the training or early in the main experiment and might then have required only minimal cues from the SSD to move in an appropriate way. However, we think that this is unlikely, for the following reasons. Firstly, seven possible predetermined locations were used (left, right, or directly ahead, at closer and further distances, and, for catch trials, the obstacle was absent). During training only a single distance was used (different to the distances used in the testing phase) and each lateral position was presented only once or twice. During the testing phase, each location was used only three times (plus six catch trials) and participants were not informed about the number of possible locations, minimizing the opportunity for learning. Consistent with this, there was no significant effect of repetition, indicating that performance did not improve over trials. Thus, we believe it is highly unlikely that participants learned the possible locations of the obstacle.

The present results are consistent with previous work suggesting that the representation of space is amodal [42], although they are also consistent with other interpretations; see [43] for a review. Blindfolded participants were able to generate a representation of space using SSD vibration information and adopt a strategy that is similar to the one used during visual navigation, as has been shown in previous work [10]. The finding that movement times and velocity corrections were greater under SSD guidance than under vision is consistent with results from a recent study [27] which showed that the number of pauses and time taken to complete a trial when navigating through virtual mazes were greater when using an SSD than when using vision. Next, we discuss possible interpretations of how participants use sensory information from a substituted modality to navigate around an obstacle.

According to representation-based control approaches [44, 45] to path planning (see [46] for a review of visually guided obstacle avoidance), participants may use the SSD to gather information about the spatial positions of the boundaries of the obstacle as they move, thus generating an internal perceptual representation similar to that obtained over a series of saccadic eye movements [47] or when combining information from a series of echolocation clicks made while moving the head [48]. Such internal representations are then used to make locomotor adjustments, similar to using visual information during locomotor actions, where representation of the body may also play a critical role [46]. The findings of the current study and of our previous study [20] suggest that, across different tasks (single obstacle circumvention and aperture navigation) and substituted modalities (haptic and auditory), internal representations based on the SSD and echoes from self-generated sound are less accurate than those based on vision. In the current study, less accurate internal representations with the SSD may have led to increased buffer space, velocity corrections and movement times. However, internal representations derived from the SSD may have been more accurate than those derived by non-expert echolocators using self-generated sounds [6].

According to the representation-based control approach, an action path is planned based on the internal representation, prior to obstacle circumvention. The greater the effective range over which the obstacle’s position can be detected, the further in advance can the path be planned, potentially allowing minimization of energy expenditure and reducing the chance of collision. In our study, vision provided an effective range covering the full length of the room (5.7 m). Thus, participants could plan their path from the start of each trial. The effective range for non-expert echolocators performing a similar obstacle circumvention task was approximately 60 cm [6]. In the current study the range of the SSD was set to 1 m, following a previous study of locomotion through apertures [20]. Under SSD guidance, participants deviated from a straight-ahead path approximately 77 cm from the obstacle. Overall, these results indicate that participants were able to plan their movements from the onset of the trial under visual guidance, whereas under SSD guidance they planned their movement once they were within the effective range of the SSD (1 m). In terms of the representation-based control approach, increasing the range of the SSD may allow participants to plan their movements further in advance, improving circumvention performance. A previous study using the EyeCane SSD with a 5 m range showed that participants were indeed able to plan their movements in a maze accurately and represent the configuration of the maze [10].

An alternative, information-based control approach [49, 50] is based on the assumption that the perceptual system uses some law of control to guide navigation on a moment-by-moment basis, using information from one or more relevant variables, such as optic flow in the case of vision [51]. Advance path planning or internal models of space are not needed to avoid an obstacle, and the variable used for control of movement may not even provide information regarding spatial layout. In our experiment, participants may have avoided the obstacle by using SSD information to inform local steering dynamics, rather than by planning the route in advance using an internal representation of space. The variable used under SSD guidance may have been less informative than the variable used under visual guidance, leading to increased buffer space, velocity corrections and movement times for the former. On the other hand, the variable used under SSD guidance may have been more informative than the variable used by the non-expert echolocating participants tested in a previous study [6], leading to faster and more accurate performance for the former.

The representation-based and information-based control approaches are not mutually exclusive, and both approaches can provide time-to-contact information. Information regarding time-to-contact to an obstacle can be obtained using visual or auditory information [52]. By monitoring the rate of change of a relevant variable such as echoic intensity when approaching an obstacle, accuracy in locating its position may increase [25, 53]. In the current study, participants could have monitored the rate of change in SSD vibration to judge their position relative to the obstacle and to avoid contacting the obstacle.

It is possible that echoic SSDs provide an aspect of vision (depth) more efficiently than echolocation alone, and for this reason the participants in the current study were able to pass on the side affording the most space in the majority of trials, whereas non-expert echolocators in a previous study [6], who did not pass on the side affording the most space in the majority of trials, did not have access to this information. The blindfolded sighted participants moved more slowly under SSD guidance than under visual guidance, but this was probably caused by the presence/absence of vision rather than to SSD use per se. Congenitally blind participants might not take longer to navigate around an obstacle with an SSD than without one, as they are used to navigating without vision.

Chebat et al. [31] showed that blind participants were better than blindfolded sighted controls at detecting and avoiding obstacles using the TDU. The improved performance of blind than of blindfolded sighted participants when using SSDs was shown to reflect cortical reorganization by Kupers et al. [54], who used the TDU to guide navigation in a virtual environment in an fMRI scanner and demonstrated that the occipital (visual) cortex was activated in congenitally blind participants but not sighted controls. The activation of the occipital cortex in blind but not sighted controls during a distance evaluation task using an echoic SSD was also reported by De Volder et al. [55]. Such cortical reorganization may result in different movement kinematics under SSD guidance between blind and sighted participants. However, this requires testing.

Peripersonal space describes space near the individual e.g. within reaching and grasping distance, and extrapersonal space is space farther from the body [4, 10, 56–58]. Sensory events in peripersonal space often require rapid motor responses such as avoidance in response to a perceived threat [56], and multisensory information may be processed differently depending on whether it is presented in peripersonal or extrapersonal space [57]. Peripersonal space can be extended or projected by altering the reaching distance [57, 58], and Chebat et al. [10] previously suggested that use of the EyeCane extended peripersonal space, contributing to success navigating through real or virtual mazes. Given the strong adaptive value of peripersonal space in detecting stimuli in close proximity to the body prior to contact [58] and the similar role of the protective envelope in obstacle avoidance during locomotion, the spatial boundary of peripersonal space may be similar to the protective envelope, and/or the buffer space measured in the current study, however this requires further investigation.

Finally, we turn to some practical aspects of the use of SSDs. In the present study, participants took approximately 11 s to move 1 m, equivalent to a walking speed of only about 0.33 km/hr. However, further training with an SSD may lead to improved performance, decreasing collisions and resulting in smoother and faster movements. Benefits of training have been shown previously. For example Kim and Zatorre [59] showed that long-term training with the vOICe SSD improved shape and pattern identification of visual images using visual-to-auditory substitution with sighted participants.

Chebat et al. [31] pointed out that the visual pattern SSD that they used to assess navigation through a maze was tested under optimal experimental conditions, and that the findings may not generalize to less ideal real-world conditions, such as when low-contrast obstacles are encountered in varied light conditions. The echoic SSD tested here was also tested under optimal experimental conditions, and caution is needed in generalizing the findings to real-world conditions, where, for example, noisy environments could reduce its effectiveness. Echoic SSDs are as effective in dark and low-level light conditions as in well-lit conditions (as are self-generated echoes used for human echolocation). However, less acoustically reflective objects may lead to lower locomotion performance based on sound echoes, while visual pattern SSDs would not be affected by acoustic reflectivity. Further investigation comparing the two classes of SSD across various environmental conditions may serve to provide greater insight regarding their applicability for practical visual rehabilitation of visually impaired individuals.

## Supporting Information

S1 File Participant data for number of collisions, side of obstacle avoidance, buffer space, overall movement times, and the number of velocity corrections for each guidance condition (SSD and vision), for each obstacle lateral location. (XLSX)
